# Modeling Gas Plasma-Tissue Interactions in 3D Collagen-Based Hydrogel Cancer Cell Cultures

**DOI:** 10.3390/bioengineering10030367

**Published:** 2023-03-17

**Authors:** Lea Miebach, Marten Hagge, Sander Bekeschus

**Affiliations:** 1 ZIK *plasmatis*, Leibniz Institute for Plasma Science and Technology (INP), Felix-Hausdorff-Str. 2, 17489 Greifswald, Germany; 2 Department of General, Visceral, Thoracic, and Vascular Surgery, Greifswald University Medical Center, Ferdinand-Sauerbruch-Str., 17475 Greifswald, Germany

**Keywords:** cancer, kINPen, redox biology, reactive oxygen species

## Abstract

Gas plasma jet technology was recently identified as a potential adjuvant in the fight against cancer. Here, the partial ionization of gas yields the local formation of an exceptional variety of highly reactive oxygen (ROS) and nitrogen (RNS) species, which are considered the main actors of plasma-induced antitumor effects. Yet, fundamental knowledge in preclinical plasma research relies on the predominant use of two-dimensional cell culture systems, despite causing significant shifts in redox chemistries that largely limit translational relevance. So far, the intricacy of studying complex plasma–tissue interactions causes substantial knowledge gaps concerning the key mechanisms and therapeutical limitations of plasma treatment in a living organism. Identifying physiologically relevant yet simplified tissue models is vital to address such questions. In our study, a side-by-side comparison of conventional and pre-established hydrogel models emphasized this discrepancy, revealing a marked difference in plasma-induced toxicity related to species distribution dynamics. Chemically embedded, fluorescent reporters were further used to characterize reactive species’ fingerprints in hydrogels compared to liquids. In addition, a thirteen cell-line screening outlined the widespread applicability of the approach while indicating the need to optimize growth conditions dependent on the cell line investigated. Overall, our study presents important implications for the implementation of clinically relevant tissue culture models in preclinical plasma medicine in the future.

## 1. Introduction

**Figure 1:**
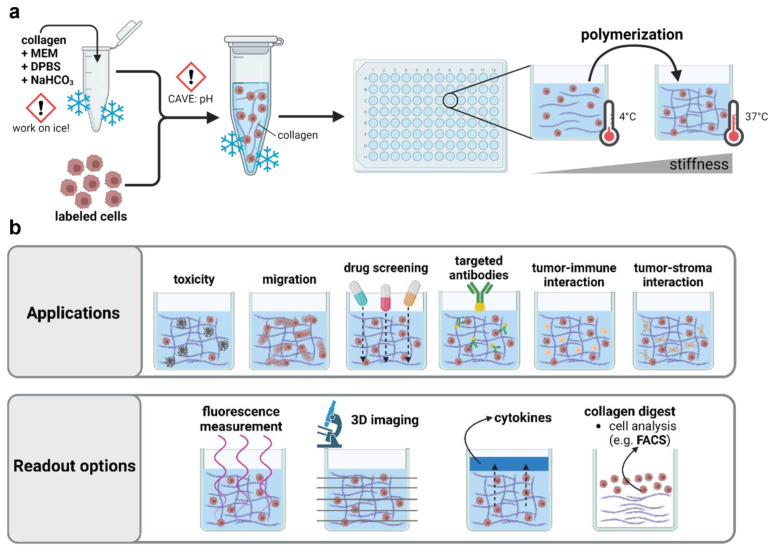
**Applications of 3D collagen-based hydrogel models in preclinical research.** (**a**) Schematic overview of collagen hydrogel preparation; (**b**) application and read-out options of three-dimensional hydrogel models. MEM = minimum essential medium; DPBS = Dulbecco’s phosphate-buffered saline; NaHCO_3_ = sodium bicarbonate.

Set in the field of applied redox biology, medical gas plasmas exploit the concept of hormesis by targeting the cellular redox state. While comparable approaches, such as photodynamic therapy, are based on the local formation of singlet delta oxygen, this novel physics-based technology is exceptional in generating a multitude of highly reactive oxygen (ROS) and nitrogen species (RNS) simultaneously. Based on the partial ionization of a noble gas, the highly reactive electrons and primary species generated in the plasma afterglow cause the formation of secondary species by energy transfer, followed by further ionization, dissociation, and excitation [1]. Initially, gas plasma therapy was accredited for application in chronic wound care [2], but the beneficial responses in the palliation of head and neck cancer patients [3], supported by numerous preclinical studies in vitro [4,5] and in vivo [6,7], outlined its potential in clinical oncology alike. A major drawback of preclinical plasma research depicts the prevailing confidence in the knowledge gained from two-dimensional cell culture systems used in the majority of studies. Despite the overall agreement that conventional cell culture models do not accurately mimic the structure, function, and physiology of living tissues, the bulk liquids surrounding cells cause significant shifts in plasma-derived ROS/RNS chemistries [8], largely limiting translational relevance. Moreover, due to the complex dynamics of plasma–tissue interaction, major mechanistic questions concerning the primary and functional, secondary penetration depths, kinetics, quenching, and influence of the target composition remain unanswered. In organic targets, charged species, such as photons and metastables, are confined to the outermost surface area, and ROS/RNS, including radical or non-radical oxygen/nitrogen species, are considered to deteriorate and react quickly [9]. Yet, medical responses have been observed in wounds [10] and (ulcerated) head and neck cancer [11] at thousands of micrometer depths, begging the question of the underlying mechanisms. While mammalian in vivo models set the benchmark for addressing the outcome and therapeutic consequences of plasma treatment in a living organism, clinically relevant tissue models are urgently needed to provide a standardized and simplified microenvironment to address such fundamental yet complex questions [12]. In the past, hydrogel-based tissue models have proven useful for cell culture purposes, as they mimic many elements of native extracellular matrices, support cell adhesion and protein sequestration, and can be easily tailored for specific applications [13,14]. In particular, collagen, as the primary constituent of native tissues, is considered an attractive material for cell studies. Hydrogel formation requires the transition of a liquid precursor solution into a solid, water-swollen network of polymers based either on noncovalent or covalent crosslinking. For instance, collagen fibrillogenesis is induced by rising temperature and pH, requiring appropriate storing conditions to prevent the spontaneous self-assembly of fibrils (Figure 1a).

Collagen-based hydrogels have been used to mimic the cellular microenvironment in a broad spectrum of studies, including mesenchymal cell differentiation and carcinoma reprogramming. Their widespread applicability outlines the attractivity to study plasma–tissue interactions in such models alike. The optical transparency of many hydrogels permits high-resolved (confocal) imaging to evaluate the effects on migration or z-resolved toxicity in three-dimensional tissue models. Complex coculture environments can be useful to address the influence and effects on tumor–stroma and tumor–immune cell interactions after plasma treatment, and cellular effects can be linked to ROS penetration depths based on embedded chemical reporters. In addition, an evaluation of the modifications at the single-cell or subcellular level by proteome or transcriptome analysis can easily be achieved after the mechanical or enzymatic harvesting of cells. (Figure 1b). However, the usage of hydrogels remains largely underscored in preclinical plasma medicine. So far, only a few studies have focused on ROS/RNS delivery in hydrogel-based model systems using embedded chemical reporters [15] or have taken advantage of culture systems with superior physiological relevance to characterize plasma-induced antitumor effects [16].

This study aimed to investigate the importance of physiologically adequate cell culture systems in life-sciences-oriented plasma research. Embedded redox-sensitive reporters were used to characterize the plasma-derived ROS/RNS chemistries in collagen-based hydrogels compared to PBS and their time-lagged release in the following. Furthermore, a side-by-side comparison of the plasma-induced toxicity in two- and three-dimensional culture systems was made. A thirteen cell-line screening indicated the widespread applicability of the approach. Overall, implementing hydrogel models in preclinical plasma research is urgently needed to identify the key mechanisms and limitations of plasma–tissue interactions in living organisms in the future.

## 2. Materials and Methods

### 2.1. Cell Culture

Thirteen cell lines were screened for their ability to grow in 3D collagen hydrogel cultures (Table 1). Prior to seeding, the cells were subcultured in *Dulbecco’s modified Eagle’s medium* (DMEM), *Roswell Park Memorial Institute* (RPMI; both Pan Biotec, Aidenbach, Germany), or *Ham’s F12K* (Thermo Fisher Scientific, Dreieich, Germany) medium supplemented with 10% fetal bovine serum, 1% glutamine, and 1% penicillin–streptomycin (all Sigma-Aldrich, Taufkirchen, Germany) according to the supplier’s instructions. The *Ham’s F12K* medium was additionally supplemented with 0.68% hygromycin (Pan Biotec, Aidenbach, Germany). The cells were kept in a specialized breeding incubator (Binder, Tuttlingen, Germany) at 37 °C, 5% CO_2_, and 95% humidity.

### 2.2. Cell Line Screening

For the prior generation of the 3D hydrogel cultures, the cells were stained with 500 nM *Vybrant DiD* cell labeling solution (Thermo Fisher Scientific, Dreieich, Germany) for 45 min at 37 °C. After washing, the cells were resuspended in 4 mg/mL collagen I (Enzo Life Sciences, Lörrach, Germany) containing 1% sodium bicarbonate (NaHCO_3_) and 19% 10× Minimal Essential Medium (MEM; Corning, Kaiserslautern, Germany), as described above. Additionally, 500 nM sytox green (SG; Thermo Fisher Scientific, Dreieich, Germany) was added for the live–dead cell discrimination. The cells were seeded in a 96-well flat-bottom plate (Greiner Bio-One, Frickenhausen, Germany) at a density of 2 × 10^5^ cells in 100 µL collagen per well and incubated for 1 h at 37 °C to induce hydrogel polymerization. In parallel, the cells were seeded in cell culture medium at a density of 1 × 10^4^ cells per well to compare the cellular growth in conventional 2D and 3D hydrogel cultures. In the 2D experiments, the number of planted cells was limited to 1 × 10^4^, as they would overgrow and consume nutrients during the growth period at higher seeding densities. The cell lines were randomly chosen in an in-house screening.

### 2.3. High Content Imaging

The cellular viability was assessed using high-content imaging (Operetta CLS; PerkinElmer, Hamburg, Germany) at 1 h and 20 h after seeding. The images were acquired in fluorescence channels at λ_ex_ 630 nm and λ_em_ 708 ± 52 nm for DiD and λ_ex_ 490 nm and λ_em_ 520 nm for Sytox Green using a 5× air (NA = 0.16) objective (Zeiss, Jena, Germany) in 8 z-dimensional planes. For the image segmentation, the z-stacks were merged into a single maximum projection image using the *Harmony 4.9*. image analysis software (PerkinElmer, Hamburg, Germany). Algorithm-driven unsupervised image analysis was performed to assess the cellular viability based on Sytox Green fluorescence.

### 2.4. Metabolic Activity

The metabolic activity of the cells cultured in the 2D and 3D models was comparatively assessed using the Resazurin Assay 20 h after cell seeding. Briefly, 7-hydroxy-3H-phenoxazin-3-on-10-oxid (resazurin; Alfa Aesar, Kandel, Germany) was added to each well at a concentration of 100 µM following incubation for 4 h at 37 °C and 5% CO_2_. Viable cells metabolize nonfluorescent resazurin into fluorescent resorufin. Fluorescence was acquired at λ_ex_ 535 nm and λ_em_ 590 nm using a multimode plate reader (F200; Tecan, Männedorf, Switzerland).

### 2.5. Plasma Source and Treatment

Plasma treatment in this study was performed using the plasma jet kINPen (neoplas, Greifswald, Germany) [17]. The jet was operated with argon (99.999% purity; Air Liquide, Bremen, Germany) at 1.5 standard liters per minute (slm) ionized at the plasma nozzle with 1 MHz and a generating power of 1-3 W. Prior to the plasma treatment, the cells were seeded in flat-bottom plates (Greiner Bio-One, Frickenhausen, Germany) at a density of 2 × 10^5^ cells in fully supplemented RPMI or 4 mg/mL collagen, as described above. After 4 h incubation at 37 °C, the cells were exposed to plasma for 60 s. The cells were incubated for another 1 h, and 200 µL fully supplemented RPMI was added on top of each well.

### 2.6. Deposition of Reactive Species

The assessment of the pH and deposition of short- and long-lived reactive species in cell-free collagen compared to PBS was conducted immediately after the plasma treatment. Briefly, collagen was prepared as described before and exposed to plasma in a 96-well flat-bottom plate (Greiner Bio-One, Frickenhausen, Germany). The relative changes in pH were assessed by adding 100 µM phenol red (Sigma-Aldrich, Taufkirchen, Germany) to collagen prior to the hydrogel polymerization. In parallel, the absolute pH determination was performed using a pH meter (Mettler-Toledo, Gießen, Germany).

**Figure A1:**
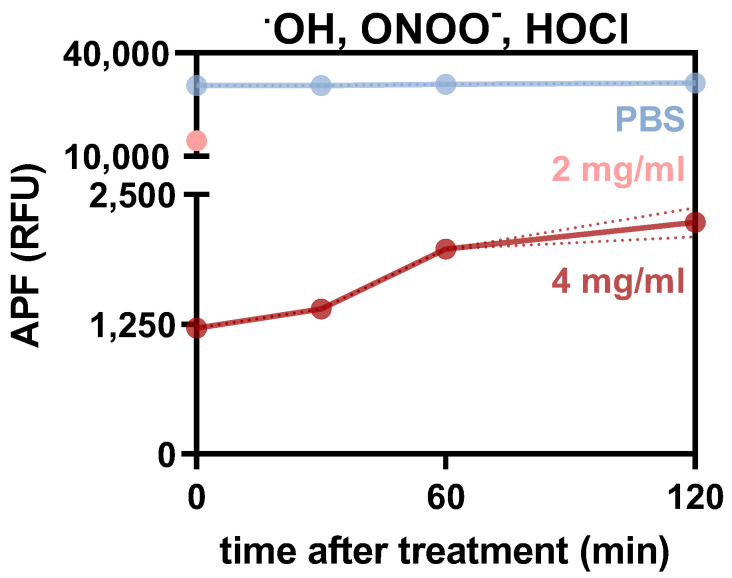
**Relative assessment of short-lived species in stiff hydrogels over time.** Relative aminophenyl fluoresceine (APF) fluorescence intensity (RFU) in PBS, 2 mg/mL and 4 mg/mL collagen 0, 30, 60 and 120 min after plasma treatment. Graph shows mean ± standard error of the mean (SEM). RFU = relative fluorescence units.

The relative assessment of the deposition of short-lived ROS into hydrogels was conducted using the redox-sensitive fluorescent probes aminophenyl fluoresceine and hydroxyphenyl fluoresceine (APF and HPF; both Enzo Life Sciences, Lörrach, Germany), which are capable of detecting hydroxyl radicals (^.^OH), peroxynitrite (ONOO^−^; both APF and HPF), and hypochlorous acid (only APF). Diaminofluoresceine (DAF; Thermo Fisher Scientific, Dreieich, Germany) was used for the detection of nitric oxide (NO^.^). All probes were added to the collagen prior to polymerization at a concentration of 5 µM. The plates were covered with aluminum foil to shield the probes from light and prevent evaporation during the polymerization. Immediately after the plasma treatment, fluorescence was determined at λ_ex_ 485 nm and λ_em_ 525 nm using a multiplate reader (F200; Tecan, Männedorf, Switzerland). In the experiments evaluating the influence of the gel stiffness on the ROS deposition, additional measurements of APF fluorescence were performed at 30, 60, and 120 min after the plasma treatment (Figure A1). The delivery of long-lived ROS/RNS was assessed indirectly. Briefly, 50 µL PBS was added on top of the gel 1 min after plasma treatment and sampled after 10 min, 20 min, 30 min, 60 min, 90 min, and 120 min. The amount of hydrogen peroxide (H_2_O_2_) was quantified using the amplex ultra red assay (Thermo Fisher Scientific, Dreieich, Germany) according to the supplier’s instructions. The fluorescence was assessed at λ_ex_ 535 nm and λ_em_ 590 n using a multiplate reader (F200; Tecan; Männedorf, Switzerland). The quantification of nitrite (NO_2_^−^) was conducted using the *Griess* Assay (Cayman Chemicals, Tallinn, Estonia) according to the manufacturer’s instructions. The absorbance was measured at 540 nm using a multimode plate reader (M200; Tecan, Männedorf, Switzerland). The ROS/RNS deposition was compared against PBS in all assays, and the absolute concentrations were calculated against a standard curve.

### 2.7. Statistical Analysis

The graphing and statistical analysis were conducted using prism 9.50 (GraphPad Software, San Diego, CA, USA). The data show the mean ± standard error of the mean (SEM), if not indicated otherwise in the figure legends.

## 3. Results

### 3.1. Basal Toxicity of Thirteen Cell Lines Grown in 3D Collagen-Based Hydrogels

**Figure 2:**
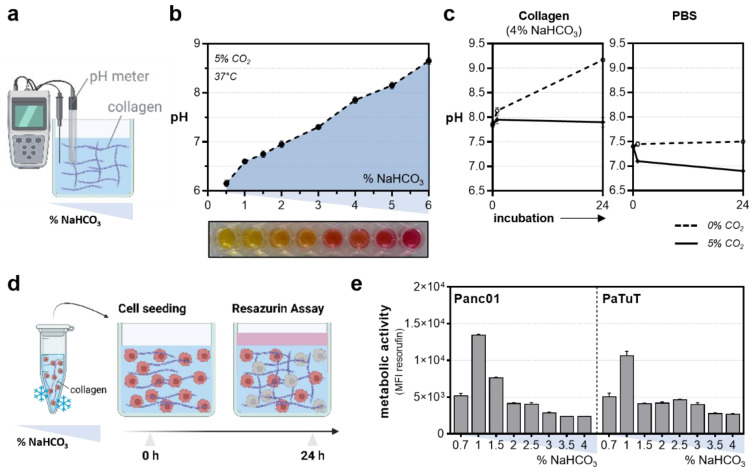
**Establishment of collagen hydrogel cultures.** (**a**) Schematic overview of the pH measurement using a pH meter; (**b**) pH levels in the different collagen formulations; (**c**) influence of the CO_2_ supply under standard incubation conditions on pH levels in a collagen-based hydrogel and PBS; (**d**) experimental procedure to optimize collagen culture conditions for cell culture purposes; (**e**) metabolic activity of Panc01 and PaTuT cells 24 h after incubation with collagen at different concentrations of sodium bicarbonate (NaHCO_3_). The graphs show the mean ± standard error of the mean (SEM). MFI = mean fluorescence intensity.

**Figure 3:**
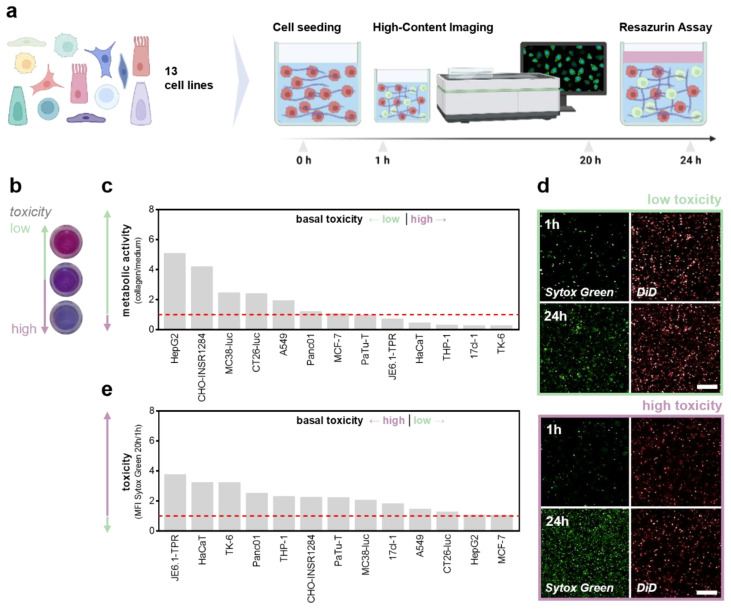
**Thirteen cell-line screening to compare basal toxicity levels in 3D collagen hydrogels.** (**a**) Schematic overview of the cell line screening; (**b**) representative images of the resazurin assay; (**c**) metabolic activity of cells grown in collagen hydrogel cultures normalized on medium controls; (**d**) representative images of sytox green and DiD labeled cells 1 h and 20 h after incubation; (**e**) sytox green fluorescence intensity after 20 h incubation normalized for 1 h. The bar graphs show the mean. Scale bar = 250 µm. MFI = mean fluorescence intensity.

The pH adjustment (Figure 2a) was optimized prior to the experiments (Figure 2b). Interestingly, the collagen-based hydrogels maintained stable pH levels upon CO_2_ supply under standard incubation conditions in the cell culture compared to the liquid buffer systems, such as phosphate-buffered saline (PBS) (Figure 2c). The assessment of the metabolic activity as a surrogate indicator of basal toxicity (Figure 2d) was performed 24 h after incubation in the different collagen formulations to ensure adequate growth conditions (Figure 2e). A thirteen cell-line screening comparing the individual growth conditions in the conventional and hydrogel-based models was conducted to evaluate the widespread applicability of the approach. Briefly, fluorescent-labeled cells were seeded in a collagen-based hydrogel containing sytox green labeling solution to identify terminally dead cells. Cellular viability was assessed immediately and 20 h after the NaHCO_3_-induced gelation using high content imaging (Figure 3a), followed by the resazurin-based evaluation of the metabolic activity (Figure 3b). Interestingly, cellular malignancy was found to be a major predictor of basal toxicity in hydrogels, indicating the need to optimize collagen formulations dependent on the cell line investigated (Table 1). While the HepG2, MC38-Luc, CT26-Luc, and A549 cells exhibited high metabolic activity when grown in three-dimensional collagen cultures, an increased basal toxicity was observed in the nonmalignant HaCaT, THP-1, 17Cl-1, and TK6 (Figure 3c). This notion was reflected by the algorithm-based unsupervised image segmentation to retrieve the number of terminally dead cells 20 h after cell seeding (Figure 3d). With minor exceptions, a higher metabolic activity was associated with low cytotoxicity and vice versa (Figure 3e).

### 3.2. Profiling Reactive Species Fingerprints in 3D Collagen-Based Hydrogels

**Figure 4:**
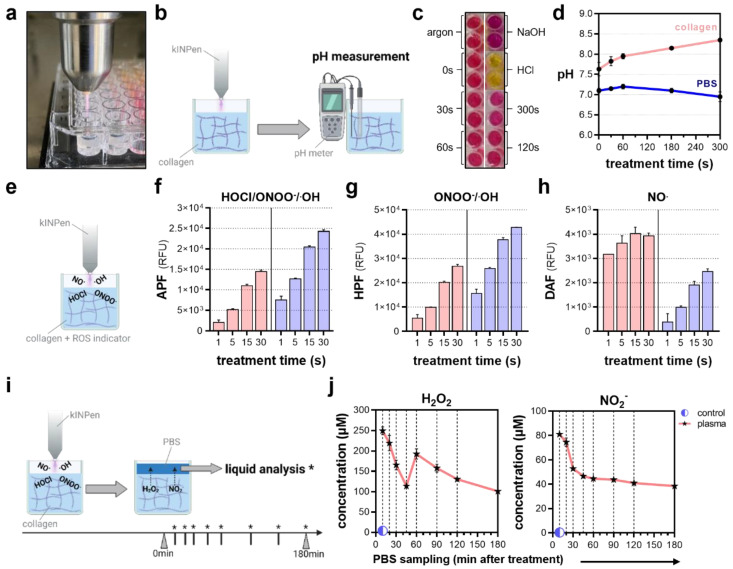
**Profiling of reactive oxygen and nitrogen fingerprints after plasma treatment.** (**a**) Representative image of collagen plasma treatment; (**b**) schematic overview of pH measurement after plasma treatment using a pH meter; (**c**) representative images of phenol red-colored collagen after plasma treatment and acidic and basic controls; (**d**) pH in collagen and PBS after plasma treatment; (**e**) schematic overview of the detection of short-lived ROS/RNS using fluorescent, redox-sensitive probes; (**f**) relative fluorescence intensity of aminophenyl fluoresceine (APF) indicative of hypochlorous acid (HOCl), peroxynitrite (ONOO^−^) and hydroxyl radicals (OH^.^); (**g**) relative fluorescence intensity of hydroxyphenyl fluoresceine (HPF) indicative of ONOO^−^ and OH^.^; (**h**) relative fluorescence intensity of diaminofluoresceine (DAF) indicative of nitric oxide (NO^.^); (**i**) schematic overview of PBS sampling to quantify the deposition and release of (**j**) hydrogen peroxide (H_2_O_2_) and nitrite (NO_2_^−^) after plasma treatment in collagen. The graphs show the mean ± standard error of the mean (SEM). * = liquid analysis was performed at this timepoint.

The intricacy of studying plasma-derived ROS dynamics delivered into a living tissue still causes fundamental knowledge gaps concerning the mechanisms and limitations of plasma–tissue interactions in vivo. Matched to the in vitro situation, the majority of studies appeal to species chemistries found in bulk liquids, showing a predominance of long-lived, secondary species generated in the liquid interphase. Recently, hydrogel models have proven useful as model systems to approach ROS/RNS delivery into tissues in vivo and were used in a side-by-side comparison with PBS in our study. Due to the generation of nitrous (HNO_2_) and nitric (HNO_3_) acid, plasma treatment is known to decrease pH levels, particularly in unbuffered solutions (Figure 4a). In contrast, pH metric evaluation of plasma-oxidized collagen (Figure 4b) revealed a slight increase in pH levels (Figure 4c) at prolonged treatment times, remaining constant in PBS as expected (Figure 4d). Embedded chemical reporters were used for the ROS/RNS profiling in collagen-based hydrogels and compared to PBS (Figure 4e). Here, a diminished deposition of peroxynitrite (ONOO^−^) and hydroxyl radicals (^.^OH) in the hydrogels was indicated by the reduced fluorescence of the redox-sensitive probes aminophenyl fluoresceine (APF; Figure 4f) and hydroxyphenyl fluoresceine (HPF; Figure 4g). Interestingly, this was contrasted by the increased fluorescence of diaminofluoresceine (DAF), which is indicative of nitric oxide (NO^.^; Figure 4h). Intriguingly, APF fluorescence was reduced to one-tenth when increasing the collagen stiffness. However, in contrast to PBS, APF fluorescence increased in the collagen over time, doubling after 120 min and eventually indicating further species formation due to the fact of tertiary reactions in the hydrogel (Figure A1). Previous studies indicated the accumulation of ROS in hydrogels followed by a time-lagged release if covered with liquids after treatment (Figure 4i). Interestingly, repeated PBS sampling over a time period of 120 min after plasma treatment revealed a bimodal release of hydrogen peroxide (H_2_O_2_). Peak concentrations were found immediately and 60 min after treatment, with a steep initial loss of approximately 150 µM in 45 min and a shallow second decline of 100 µM in 2 h. Monitoring nitrite (NO_2_^−^) release dynamics showed a steep decline in the first 45 min alike, while the measured concentrations remained largely constant at 40 µM over the next 75 min (Figure 4j).

### 3.3. Differential Plasma Sensitivity in Three-Dimensional Hydrogel Models

**Figure 5:**
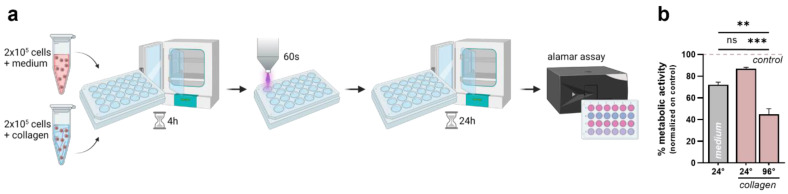
**Differential plasma sensitivity of 3D cultured cells compared to conventional 3D culture systems.** (**a**) Schematic overview of the experimental procedure; (**b**) metabolic activity of cells cultured in medium or collagen and different plate geometries 24 h after plasma treatment. The bar graphs show the mean ± standard error of the mean (SEM). The statistical analysis was performed using one-way analysis of variance (** *p* < 0.01; *** *p* < 0.001).

Finally, a side-by-side comparison was made to characterize the antitumor efficacy of plasma treatment in two- and three-dimensional culture systems. Briefly, 2 × 10^5^ cells were seeded in medium or collagen-based hydrogels in a 24-well flat bottom plate. In addition, different plate geometries were tested to address the differences in species distribution in the solid hydrogels. After 4 h of incubation, the cells were exposed to plasma for 60 s. The cellular metabolic activity was evaluated after 24 h using the alamar blue assay (Figure 5a). Plasma-induced toxicity was partially reduced when the cells were cultured in collagen in 24-well plates. Surprisingly, the cellular metabolic activity decreased to 40% when the cells were growing in 96-well plates, indicating a major impact of the plate geometry (Figure 5b).

## 4. Discussion

In a groundbreaking study, Peterson and colleagues demonstrated that healthy mammary epithelial cells display tumorigenic potential when cultured in conventional monolayer culture but form multicellular structures that resemble healthy acini when grown in three-dimensional hydrogels [18]. Supported by intensive investigations in many other biological research areas [19,20], these findings emphasized the notion that cells behave more natively when cultured in three-dimensional environments and revealed a considerable bias concerning cellular susceptibility in drug screening studies [21]. Increased awareness of this topic has forwarded the development of novel bioengineered models supporting organization and differentiation as found in living tissues to increase translational research in vitro.

The use of adequate and clinically relevant model systems becomes particularly apparent when investigating multimodal approaches such as medical gas plasmas. This novel physics-based technology is exceptional in generating a variety of highly reactive species simultaneously based on the partial ionization of a noble gas. Exploiting the concept of hormesis, the generated species are considered to target the cellular redox state, inducing oxidative eu- or distress [22] dependent on the applied dose. Following this, the initial research in the field of plasma medicine focused on its applicability in chronic wound care [10], leading to the successful development of several marketed plasma devices that are approved as medical devices and regularly applied in clinical dermatology [17,23]. Strikingly, clinical case studies intended to reduce the microbial load and unpleasant odor in patients suffering from advanced therapy refractory head and neck carcinoma outlined the potential of medical gas plasmas in clinical dermato-oncology in 2015. Despite increasing the patients’ life quality, plasma treatment induced tumor cell apoptosis and partial remission in one-third of patients [3,24]. These findings have been supported by intensive investigations emphasizing the antitumor efficacy of plasma in numerous tumor models in vitro and in vivo in recent years. As a major drawback, many assumptions have been made based on conventional cell culture models in vitro, disregarding shifts in the redox chemistries [8] and the rapid consumption of species occurring in biological tissues [25] compared to diffusion systems such as liquids [26]. The lifetimes of biologically relevant, plasma-derived species, such as ^.^OH, ONOO^−^, O_2_^−^, and HO_2_, range from nanoseconds to a few seconds, questioning the effective penetration depths in biological systems [27,28,29] and limiting widespread investigations. Moreover, fundamental mechanistic questions concerning the primary and functional, secondary penetration depths, kinetics, quenching, and influence of the target composition remain unanswered as of now. Standardized, simplified in vitro models are urgently needed to address such fundamental yet complex questions, which cannot be achieved in 2D culture systems conventionally used in preclinical plasma research. The limited transferability remains a major gap in redox-focused plasma research.

So far, ROS penetration depths have been predominantly studied in hydrogels as tissue model systems or as a tissue barrier above liquids containing redox-sensitive reporters. In 2014, Szili and colleagues reported penetration depths in the range of 150 µm to 1.5 mm after 10 min plasma treatment by adding a gelatin film over liquids containing OPD/HRP biological sensors in a cuvette [15]. Later, comparable results were obtained in the same group, using a 1 mm pig skin layer as a natural tissue barrier in a similar setup [30]. Directly assessing species penetration in hydrogels has been conducted using the relatively insensitive potassium iodide starch assay [31] or various fluorescent reporters [16]. The relative deposition rates of short-lived species were found to be reduced in our study when compared to PBS, conceivably indicating a consumption as discussed above. Interestingly, differential results were found concerning increased levels of long-lived H_2_O_2_ and NO_2_^−^, reported by Labay and colleagues in 2020. As seen in our study, the accumulation and time-lagged release of ROS even motivated the use of plasma-treated hydrogels as a therapeutical ROS depot for treating internal tumor lesions [32,33]. In vivo, direct evidence of increased intratumoral ROS levels post plasma treatment has been provided by intravenous injection of a redox-sensitive, luminol-based probe into tumor-bearing mice revealing a local increase in the luminescence compared to the untreated controls [30,34].

Due to the complexity of plasma–tissue interactions, the majority of studies attempted to characterize the functional rather than primary ROS penetration depth based on tissue sectioning to assess tissue apoptosis, molecular effects by Raman spectroscopy ex vivo [35,36,37], or relative evaluation of blood flow and oxygenation assessed by hyperspectral imaging in mice and human skin. Importantly, the tissue’s histological structure is considered to have a major impact on the penetration of species in deeper layers. While nonkeratinized tissues have shown effects up to a few hundred micrometers [38], plasma effects may not reach much beyond the stratum corneum in keratinized skin [39]. However, the secondary effects, including paracrine cell signaling events, have been suggested to still occur at layers several millimeters deep into the tissue [40,41], but the mechanisms remain unclear. In the future, hydrogel models could help to address such fundamental questions, for instance, using z-resolved high content imaging [16]. The importance of implementing the use of physiologically relevant culture systems was emphasized by the partially reduced sensitivity of the cells grown in hydrogels in our study. Comparable results have recently been reported in drug-screening studies [21]. However, the plasma efficacy in hydrogels was surprisingly influenced by the plate geometry, and the toxicity increased up to 50% when the cells were seeded in 96-well compared to 24-well plates. Considering a reduced local retention time in the 24-well plates due to the fact of an increased surface area that needs to be covered, and this remarkable difference outlines the discrepancy in species distribution in liquids as diffusion systems compared to solid, tissue-like gels.

As a natural material, collagen-based hydrogel cultures have some important drawbacks, including low stiffness, limited long-term storability and batch-to-batch variability. In our study, the nonmalignant HaCat, 17cl-1, THP-1, and TK6 cells showed higher basal toxicity than the malignant cells. Hydrogel optimization was performed using two malignant cell lines, showing favored growth conditions at collagen pH levels slightly below pH 7.4, which is the physiological pH in blood and interstitial fluids. Particularly, the blood-derived THP-1 cells adapted to the environment, providing strong buffer systems assuring only marginal changes in the pH. By contrast, due to the fact of metabolic shifts leading to increased lactate formation [42], tumor cells are known to reside in a slightly acidic environment that is considered to promote angiogenesis, suppress antitumor immune responses, and increase their metastatic potential [43,44]. Hence, optimizing hydrogel formulations for individual cell-type-specific applications might be useful.

Overall, our study emphasizes the use and widespread applicability of three-dimensional hydrogel models in plasma research. Implementing physiologically relevant cell culture models will help increase the translational relevance of preclinical research in the future and gain novel insight into the limitations and mechanisms of plasma-induced toxicity observed in vivo.

## 5. Conclusions

Collagen-based hydrogel models were investigated for their ability to characterize plasma–tissue interactions in preclinical studies. Embedded redox-sensitive reporters were used to characterize plasma-derived ROS/RNS chemistries in collagen-based hydrogels compared to PBS and their time-lagged release in the following. A side-by-side comparison of the plasma-induced toxicity in two- and three-dimensional culture systems revealed a diminished plasma susceptibility of the cells when grown in hydrogels. A major influence of the plate geometry indicated the impact of the different species distribution in liquids as diffusion systems compared to solid, tissue-like gels. Moreover, a cell line screening done to assess the basal toxicity of thirteen different cell lines grown in hydrogels outlined the widespread applicability of the approach.

## Figures and Tables

**Table 1:** **Cell lines.** Overview of the cell lines, their species and organ origin, supplier, and corresponding results from metabolic activity (ratio; color gradient indicates a high (green) or low (white) basal metabolic activity in collagen hydrogels compared to conventional cell culture) and sytox green (SG; color gradient indicates a high (red) or low (white) basal toxicity in collagen hydrogels compared to conventional cell culture) assays. ca = malignant cell line; lymph = lymphocyte; hu = human; Mu = murine; ha = hamster; Ref-No. = reference number.

Cell Line	Origin	Species	Supplier	Ref-No.	Ratio	SG
17Cl-1	fibroblasts	mu	BEI_Resources	NR-53719	0.30	1.85
A549	lung (ca)	hu	ATCC	CCL-185	1.96	1.49
CHO	ovary	ha	DMSZ	ACC 858	4.22	2.27
CT26-luc	colon (ca)	mu			2.43	1.30
HaCaT	keratinocytes	hu	ATCC	PCS-200-011	0.48	3.26
HepG2	hepatocytes (ca)	hu	ATCC	HB-8065	5.11	1.10
JE6.1-TPR	T lymph (ca)	hu	MedUni Wien	-	0.74	3.79
MC38-luc	colon (ca)	hu			2.49	2.08
MCF7	mamma (ca)	hu	ATCC	HTB22	1.10	1.08
Panc01	pancreatic (ca)	hu	ATCC	CRL-1469	1.23	2.54
PaTuT	pancreatic (ca)	hu	DMSZ	ACC 162	1.01	2.25
THP-1	monocytes	hu	DMSZ	ACC16	0.34	2.32
TK6	lymphoblast	hu	CLS	300357	0.30	3.25

## Data Availability

The underlying data of this study are available from the corresponding author upon reasonable request.
